# Screening and brief interventions for hazardous and harmful alcohol use among patients with active tuberculosis attending primary care clinics in South Africa: a cluster randomized controlled trial protocol

**DOI:** 10.1186/1471-2458-11-394

**Published:** 2011-05-26

**Authors:** Karl K Peltzer, Pamela P Naidoo, Gladys G Matseke, Khangelani K Zuma

**Affiliations:** 1 HIV, AIDS, TB, and STIs (HAST), Human Sciences Research Council (HSRC), Pretoria, South Africa; 2 Department of Psychology, University of Limpopo, Turfloop, South Africa; 3 HIV, AIDS, TB, and STIs (HAST), Human Sciences Research Council (HSRC), Cape Town, South Africa; 4 Department of Psychology, University of the Western Cape, Cape Town, South Africa

## Abstract

**Background:**

In 2008 the World Health Organization (WHO) reported that South Africa had the highest tuberculosis (TB) incidence in the world. This high incidence rate is linked to a number of factors, including HIV co-infection and alcohol use disorders. The diagnosis and treatment package for TB and HIV co-infection is relatively well established in South Africa. However, because alcohol use disorders may present more insidiously, making it difficult to diagnose, those patients with active TB and misusing alcohol are not easily cured from TB. With this in mind, the primary purpose of this cluster randomized controlled trial is to provide screening for alcohol misuse and to test the efficacy of brief interventions in reducing alcohol intake in those patients with active TB found to be misusing alcohol in primary health care clinics in three provinces in South Africa.

**Methods/Design:**

Within each of the three selected health districts with the highest TB burden in South Africa, 14 primary health care clinics with the highest TB caseloads will be selected. Those agreeing to participate will be stratified according to TB treatment caseload and the type of facility (clinic or community health centre). Within strata from 14 primary care facilities, 7 will be randomly selected into intervention and 7 to control study clinics (42 clinics, 21 intervention clinics and 21 control clinics). At the clinic level systematic sampling will be used to recruit newly diagnosed TB patients. Those consenting will be screened for alcohol misuse using the AUDIT. Patients who screen positive for alcohol misuse over a 6-month period will be given either a brief intervention based on the Information-Motivation-Behavioural Skills (IMB) Model or an alcohol use health education leaflet.

A total sample size of 520 is expected.

**Discussion:**

The trial will evaluate the impact of alcohol screening and brief interventions for patients with active TB in primary care settings in South Africa. The findings will impact public health and will enable the health ministry to formulate policy related to comprehensive treatment for TB and alcohol misuse, which will result in reduction in alcohol use and ultimately improve the TB cure rates.

**Trial registration number:**

PACTR: PACTR201105000297151

## Background

The 2008 World Health Organization (WHO) Report found that South Africa had the highest tuberculosis (TB) incidence in the world, at over 5 times the average incidence rate found in the 22 high-burden countries. In 2006, South Africa with only 0.7% of the world's population had an estimated 28% of HIV positive adult TB cases reported globally. Over the last 5 years TB case notification has increased by a massive 81%, from 188 695 cases in 2001 to 341 165 cases in 2006. In 2006 KwaZulu-Natal, one of the nine provinces in South Africa, had the highest total TB caseload accounting for 31% of all TB cases nationally [1].This increase is mostly associated with the co-morbidity between HIV and TB. However, other factors, such as poverty and alcohol misuse is also associated with TB incidence. The role of alcohol misuse in increasing TB incidence has been under-studied within the South African context and has consequently not been adequately addressed in TB prevention efforts.

Excessive alcohol use has been causally linked to TB incidence. Two pathways are involved: the first is biological via weakening of the immune system, and the second is social via social exclusion and drift, resulting in about a threefold increased risk of TB [2]. Alcohol use was estimated to have been responsible for 939 000 disability-adjusted life-years lost in South Africa for TB and HIV/AIDS alone in 2004 (253 000 for women, 687 000 for men). This figure corresponds to 4.6% of the overall disease burden in South Africa (2.5% for women, 6.6% for men). These numbers show the potential for reducing alcohol-attributable infectious disease burden in South Africa, since cost-effective measures for reducing alcohol-attributable harm in developing societies exist and could be applied [2].

There are numerous studies cited in the literature that support the strong association between alcohol use, alcohol use disorders and TB [3-8]. Numerous studies show pathogenic impact of alcohol on the immune system causing susceptibility to TB among drinkers [4,7,9]. "Alcohol use strongly influences both the incidence and the outcome of the disease and was found to be linked to altered pharmacokinetics of medicines used in the treatment of TB, social marginalization and drift, higher rate of re-infection, higher rate of treatment defaults and development of drug-resistant forms of TB; about 10% of the TB cases globally were estimated to be attributable to alcohol" [3]. People that drink heavily show higher relapse rates, a higher probability of an unfavourable clinical course and a higher probability of experiencing the most destructive forms of TB. High prevalence of alcohol misuse in most population groups have been reported in South Africa. A household survey conducted in Mamre in the Western Cape Province (a community of approximately 5 000 people) found a positive association between TB and alcohol problems in the households [10].

Hazardous and/or harmful alcohol use is on the increase in developing/middle income countries including South Africa. Hazardous drinking is defined as a quantity or pattern of alcohol consumption that places patients at risk for adverse health events, while harmful drinking is defined as alcohol consumption that results in adverse events (e.g., physical or psychological harm) [11]. In South Africa more than 22% of men and women engage in hazardous or harmful drinking during weekends [12]. Among a primary health care outpatient sample of 600 rural South Africans 37.4% of men and 10.7% of women were found to be hazardous drinkers, and 9.2% of men and 0.3% of women meet criteria for probable alcohol dependence or harmful drinking as defined by the AUDIT [13]. Prevalence estimates range from 4% to 29% for hazardous drinking and from less than 1% to 10% for harmful drinking [11]. In a another multi-country study a prevalence of hazardous alcohol use of 18% (after non-drinkers and alcoholics had been excluded) was found among patients attending primary health care facilities in Australia, Bulgaria, Kenya, Mexico, Norway and the USA [14]. Similar prevalence rates of hazardous drinking in primary care outpatients were found in Nigeria (28.6%) [15] and 25% in Harare, Zimbabwe [16], and a low prevalence of above 1.7% alcohol dependency or harmful drinking in Nigeria [17].

In March 2006, in line with a WHO/AFRO decision of African Health Ministers, a TB Crisis Management Plan was launched by the Minister of Health in South Africa. The plan focused on 4 districts in South Africa which had a quarter of the national TB case load. These districts were: Amatole and Nelson Mandela Metro in the Eastern Cape Province, EThekwini Metro in KwaZulu-Natal Province, and the Johannesburg Metro in Gauteng Province. The mainstay of the plan was social mobilization to ensure that TB is de-stigmatized, that people seek treatment early, and that patients complete treatment [18].

### Screening and brief interventions for alcohol use disorders

Increasing emphasis has been placed on the detection and treatment of hazardous and harmful drinking disorders, particularly among patients who are seen in primary health care settings [11]. Screening instruments such as the Alcohol Use Disorders Identification Test (AUDIT), CAGE, and Screening and Brief Interventions (SBIs) have been found to be useful in detecting and treating alcohol use disorders in a number of settings [13,19,20]. The interventions are based on cognitive- behavioural interventions and motivational interviewing techniques and have been found to be effective and efficient in the treatment of alcohol use disorders in most chronic conditions. In previous studies screening and brief intervention for alcohol problems have been successfully implemented by nurses in demonstration projects in South Africa as part of a WHO strategy to expand screening and brief intervention for alcohol problems in developing countries funded by WHO and NIAAA [13,19]. Community health workers have been identified as strategic implementation agents for screening and brief intervention of alcohol problems in primary care in South Africa.

Whilst there have been studies conducted on screening for alcohol misuse and brief interventions producing favourable results, there is a dearth of scientific literature on evidence-based best practice methods to screen for alcohol misuse and brief interventions amongst individuals with active TB. Given the fact that in South Africa the target rate for TB cure has not been met, and the fact that alcohol use and misuse is known to be a causal factor in TB onset, there is an urgent need to conduct a cluster randomized control trial to evaluate SBIs for alcohol use disorders among TB patients. This should be perceived as a key intervention to improve the outcomes for TB treatment and control. The alcohol-related risk reduction intervention programme proposed in this study, will be based on a modified Information Motivation and Behavioural Skills (IMB) model of health promoting behaviours and alcohol risk reduction intervention to reduce hazardous and/or harmful alcohol consumption.

### Aim of the study

The aim of this study is to conduct a cluster randomized control trial to assess the effectiveness of SBI for alcohol use disorders among TB patients. Consenting patients who are starting TB treatment and screen for alcohol use risk are randomized, with the primary care clinic being the unit of randomization into one of two arms: The first arm being a Brief Intervention for alcohol misuse arm (treatment arm) and the second arm being the treatment as usual where patients receive an alcohol education leaflet (control arm).

#### Objectives

1. To screen for alcohol misuse among TB patients in the selected sites.

2. To implement Screening and Brief Interventions (SBI) among TB patients that screen positive for alcohol misuse.

3. To monitor and evaluate the treatment outcomes for both alcohol misuse and TB among TB patients.

4. To report on the outcome of the intervention (SBI) and make recommendations for future interventions for reduction in alcohol use.

## Methods/Design

### Setting

Three provinces, in South Africa, with the highest TB caseload will be selected for inclusion in the study. One district in each province (N = 3) with the highest TB caseloads will ultimately be included. These districts are Siyanda in Northern Cape Province, Nelson Mandela Metro in the Eastern Cape Province, and EThekwini in KwaZulu-Natal Province. Within each of these three study districts 14 primary health care facilities will be selected on the basis of the highest TB caseloads per clinic (N = 42). The type of health facility will be a primary health care clinic or community health centre. The study catchment areas within the study health districts and randomization procedures will enable broad coverage of major population groups.

### Design

In order to assess the effectiveness of the Screening and Brief Interventions (SBI) among participants newly diagnosed with TB and found to be misusing alcohol, a cluster randomized controlled trial design will be implemented. All new TB patients will be screened using the Alcohol Use Disorder Identification Test (AUDIT). TB patients who meet the cut-off for misusing alcohol both in the intervention and control arms will be reassessed after baseline assessment at time 2 (3 months following intervention) and time 3 (6 months following intervention). The intervention will comprise the following: personalized feedback on AUDIT results, a health education leaflet, simple advice and brief counselling about reducing excessive drinking, during one -20 minute- session. The trial will incorporate cluster randomization of primary health care facilities to avoid the risk of contamination.

#### Study hypotheses

• Individuals receiving the brief intervention will experience a greater change in mean number of AUDIT (alcohol risk) scores and mean number of heavy drinking days, compared with the individuals receiving a health education leaflet only (control group)

• Alcohol consumption (use and misuse) will be associated with anti-tuberculosis medication adherence and TB treatment outcomes.

### Principles for recruitment

#### Inclusion criteria

##### Primary Health Care Clinics

Clinics with a high TB case-load (based on statistics collected by the Department of Health) in each of the three study districts with a high burden of TB will be included in the study.

##### Patients with active TB

New tuberculosis treatment patients (i.e. those patients who have been initiated or have been on anti-TB treatment for less than one month), males and females, 18 years and above who visit the primary health care facility and who score 8 or more for men and 7 or more for women on the AUDIT questionnaire after screening will be included in this study. Some researchers found that brief interventions are effective even among excessive drinkers [21-23]. Therefore patients scoring high on the AUDIT are included in the study.

#### Exclusion criteria

##### Patients with active TB

New tuberculosis treatment patients under the age of 18 years will not be included. In addition those individuals 18 years and older scoring less than 8 for men and less than 7 for women on the AUDIT questionnaire will be excluded from the study. Women who are pregnant, patients already under treatment for alcohol misuse and patients who have symptoms of psychosis and who are mentally handicapped will be excluded.

#### Randomisation

Randomisation will be conducted using a secure remote randomization service. Within each district in each of the three provinces the 14 primary health clinics with the highest TB caseloads will be randomly assigned to the treatment and control arms, stratified by clinic type (clinic and community health centre) and TB case load. TB patients misusing alcohol will be randomized to either the treatment or control group. At clinic level all consecutive new TB patients will be systematically recruited over a period of six months.

#### Blinding

Participants (clinic staff members and TB patients) will not be blind to their intervention or delayed intervention status. However, to protect against information biases in the reporting of alcohol use and TB adherence behaviour, the data collection team who will assess the outcomes will be blind to the clinic's status as intervention or delayed intervention arm.

### Procedure

**Figure 1:**
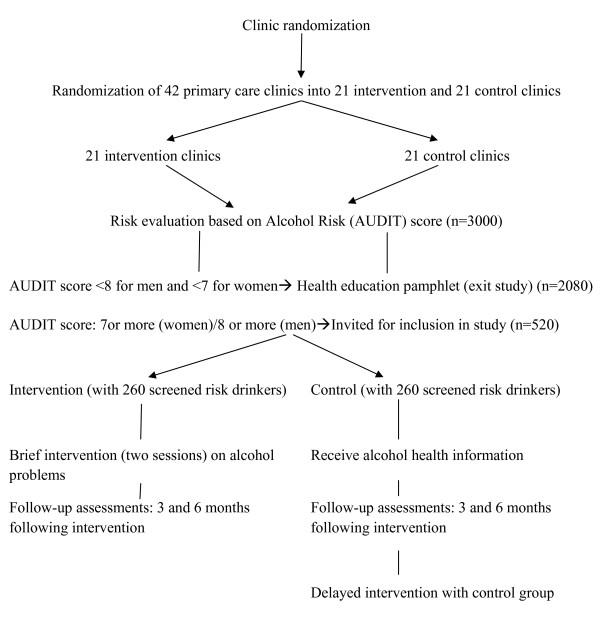
**Overview of study activities**.

Universal screening of all new tuberculosis treatment patients will be used whereby all consecutive clients visiting the primary health care facility will be screened for alcohol problems and be offered a brief counselling intervention. A health care provider who identifies a new TB treatment patient (within one month on treatment) will inform the patient about the study and refers the patient for participation if interested. A research assistant will ask for permission/consent from patients attending the primary care facility to participate in the stage 1 of the study, i.e. screening or baseline assessment using the AUDIT questionnaire. This will take about 15 minutes. The research assistant will not be involved in delivering the brief counselling intervention. All participants undergo the initial assessment and the research assistant will score the results of the alcohol test section of the questionnaire. Tuberculosis patients who will score 8 or more for men and 7 or more for women on the AUDIT questionnaire after screening (risky drinkers) will then be approached by the research assistant for a second informed consent for enrolment in stage 2, the intervention study. For patients included in the study, the research assistant refers the patient to a clinic counsellor who carries out the intervention (experimental or control) for all the participants after which they will be followed up at 3 months and 6 months at the health facility, and assessments will be done by the research assistant. The experimental intervention includes a brief counselling intervention on alcohol risk reduction consisting of two sessions, the first immediately after alcohol screening and the second within a month thereafter. In the control condition the clinic counsellor provides an alcohol education leaflet, and after completion of the 6 months follow-up, the trained clinic counsellor will provide the delayed intervention of brief counselling intervention on alcohol risk reduction. At follow-up at least six individual attempts are made to contact patients by telephone and letter. Even if a contact was not successful at 3 months, further attempts will be made at 6 months. Sampling will occur throughout all hours of clinic operation over a 6 months period. Thirteen tuberculosis patients will be recruited from each primary health care facility (n = 13). Participants will receive Rand 60 (8.5 US$) transport reimbursement at 6-month follow-up (see Figure 1).

#### Screening

The 10-item Alcohol Disorder Identification Test (AUDIT) [24] assesses alcohol consumption level (3 items), symptoms of alcohol dependence (3 items), and problems associated with alcohol use (4 items). Responses to items on the AUDIT are rated on a 4-point Likert scale from 0 to 4, for a maximum score of 40 points. Higher AUDIT scores indicate more severe levels of risk: a score of 8 indicates a tendency for problematic drinking. To comply with the timeline of this study, all subjects were asked for their alcohol consumption in the previous 3 months rather than in the past 12 months.

The primary outcome measure used in this study is the change in drinks per week from baseline to follow-up, as estimated from the first two AUDIT questions. A secondary measure is the frequency of consuming four or more drinks per occasion (AUDIT question 3). We will also evaluate the percentage of patients whose weekly consumption decreases, increases, or remains the same, as well as changes in a combination of the first three AUDIT item scores called the Drinkers' index, in order to estimate the amount of alcohol risk reduction.

#### Consent

Consent to participate will be obtained in a 2-stage process. Research assistants will initially ask for informed consent to conduct a health screen and collect basic information and check eligibility to take part. No identifiable information will be collected at this stage. Patients who have a positive score on the AUDIT (alcohol risk score), as applicable, will have the study explained to them verbally and in writing (using the patient information sheet). Informed consent will be obtained at this second stage which will include permission to give the contact details to the research staff, and participate in the experimental or control condition and follow up after 3 and 6 months by the research assistant.

#### Interventions

##### Health Education Leaflet

All the patients randomized and allocated to the control group will complete the baseline measures and will receive a health education leaflet on responsible drinking.

##### Brief Counselling

The patients randomized and allocated to the intervention arm will complete baseline measures and receive brief counselling for alcohol reduction intervention. The goals for brief counselling are as follows: 1) To identify any alcohol- related problems mentioned in the interview, 2) To introduce the sensible drinking leaflet, emphasis the idea of sensible limits, and make sure that patients realize that they are in the risk drinking category, 3) To provide feedback on the relationship between alcohol and TB treatment [25,26], 4) To work through the first 3 sections of the problem solving manual while mentioning the value of reviewing the other sections, 5) To describe drinking diary cards, 6) To identify a helper, and 7) To plan a follow-up counselling session.

The Information-Motivation-Behavioural Skills (IMB) Model will be used in the study to guide the alcohol reduction intervention. The IMB model [27-29] proposes that *information *about alcohol misuse and methods of reducing and preventing harmful and/or hazardous drinking is a necessary precursor to risk reduction. *Motivation *to change, however, also directly affects whether one acts on information about risk and risk reduction. Finally, the IMB model holds that *behavioural skills *related to preventive actions represent a final common pathway for information and motivation to result in alcohol risk behaviour change. The IMB model posits that information and motivation activate behavioural skills to ultimately enact risk reduction behaviours. The IMB model also shows that information or motivation alone can have direct effects on some preventive behaviour, such as when information about risky alcohol drinking prompts drinking in moderate levels or to stop drinking.

However, behavioural skills become increasingly important when preventive actions require complex skills such as requesting that a family member, friend, or patient to reduce amount of alcohol, avoiding alcohol use in sexual contexts, or to stop drinking. Thus risk reduction skills are theorized to have a direct effect on risk reduction behaviours, and information and motivational enhancement may have direct effects as well as indirect effects on risk reduction behaviours. The content of brief interventions has varied somewhat in research and demonstration projects. Most programmes are instructional and motivational, designed to address the specific behaviour of drinking with information feedback, health education, skill-building and practical advice, rather than with psychotherapy or other specialized treatment techniques [24].

##### Assess and Tailor Advice to Stage of Change

Further assessment beyond initial screening can be an important aid to brief counselling. Diagnostic assessment involves a broad analysis of the factors contributing to and maintaining a patient's excessive drinking, the severity of the problem, and the consequences associated with it. Another type of assessment is the motivational stage of the patient, which can vary from no interest in changing drinking behaviour (pre-contemplation) to actual initiation of a drinking moderation plan (action stage). The Stages of Change represent a process that describes how people think about, initiate, and maintain a new pattern of health behaviour. The five stages are each matched with a specific Brief Intervention element. One of the simplest ways to assess a patient's readiness to change their drinking is to use the "Readiness Ruler" recommended by Miller. Ask the patient to rate on a scale of 1 to 10, "How important is it for you to change your drinking?" (with "1" being not important and "10" being very important). Patients who score in the lower end of the scale are pre-contemplators. Those who score in the middle range (4-6) are contemplators, and those scoring in the higher range should be considered ready to take action. It is helpful to begin counselling in a way that meets the patient's current motivation level. For example, if the patient is at the pre-contemplation stage, then the advice session should focus more on feedback in order to motivate the patient to take action. If the patient has been thinking about taking action (contemplation stage), emphasis should be placed on the benefits of doing so, the risks of delaying, and how to take the first steps. If the patient is already prepared for taking action, then the health worker should focus more on setting goals and securing a commitment from the patient to cut down on alcohol consumption. For most patients, the standard sequence of Feedback, Information, Goal Selection, Advice, and Encouragement should be followed, with minor modifications dictated by the current stage of change.

##### Using the tuberculosis illness episode to engage the patient

The content of the brief counselling intervention includes normative feedback on the patient's alcohol consumption, including encouraging the patient to reflect on how drinking may be impacting his or her health. Incorporating feedback on the relationship between alcohol and TB treatment is an important component of the intervention. Counsellors provide general information on the negative consequences of alcohol misuse on TB treatment outcomes and TB treatment adherence, exploring the association between drinking and the risk of unsuccessful TB treatment [25].

##### Provide Skills Training via the Self-Help Bookle

After assessing the patient's readiness to change drinking behaviour, the provision of a self-help booklet is recommended. A modified version of the self-help manual used in the WHO Project on Identification and Management of Alcohol-Related Problems as adapted to South Africa has been developed by [30].

### Counsellor training and intervention quality assurance

The intervention counsellors will consist of existing lay counsellors and nurses from the study clinics speaking the predominant languages in the area (English, Afrikaans, Zulu, Xhosa and Tswana) and they will deliver the interventions to men and women as per usual clinic services. All lay counsellors and up to four nurses per study clinic who are suitable to deliver the brief counselling intervention will receive formal training (lay counsellors 3 days and nurses 2 days) and supervision. The training takes a practical, systems approach, aiming to facilitate the implementation of SBI in clinic operations rather than merely educating staff. The training curriculum contains modules addressing practical issues deemed essential to implementing the programme. For early identification of alcohol problems in primary care the Alcohol Use Disorders Identification Test (AUDIT) [24] and for the brief intervention the WHO brief intervention package for hazardous and harmful drinking [24] will be used. Both were adapted to the South African context, e.g. in terms of standard unit of alcoholic drink and drinking limits. The AUDIT was translated and back translated according to scientific standard procedures [31] into four of the major languages (Tsonga, Northern Sotho, Venda, and Afrikaans) [13] and will be translated into additional languages (Xhosa, Zulu and Tswana). The self-help booklet for patients and a handout on "cutting back" showing the drinking limits and health effects of risky alcohol consumption were also made available in the target languages. The AUDIT manual explains the purpose of screening for alcohol problems in primary care, the context of alcohol screening, the development and validation of the AUDIT, administration guidelines, scoring and interpretation. The Brief Intervention manual defines concepts and terms, roles and responsibilities of Primary Health Care, SBI: a risk management and case finding approach, alcohol education for low-risk drinkers, abstainers and others, and simple advice an brief counselling for risk zone drinkers, self-help booklet and training sources. Critical administrative activities include administration and scoring of the screening instruments, assuring availability of patient brochures, sequencing of interventions with treatment of presenting health problems, the essential elements of an intervention, and the management of SBI records.

The training will comprise of four elements: orientation to the relevant practice, standardised PowerPoint presentation, tape recorded simulated consultations with trained actors and ongoing clinical supervision by experienced HSRC staff. The simulated consultations will be recorded and rated by two independent clinical assessors. The brief intervention counsellor will be assessed for adherence to the brief counselling protocol in addition to their behaviour and skills using a Behaviour Change Counselling Index. Assessors will submit the ratings, comments and supervision points for each consultation. This information will support clinical supervision and training until the brief intervention counsellor reaches a required standard of practice agreed by the assessors [32,33].

To help protect against counsellor drift, the brief intervention will be completely manualized and can be used to guide the counsellor through the session content. Site visits will be done bi-weekly by the project manager to offer support and supervision to the trained clinic based study fieldworkers. In terms of control around the quality and consistency of the implementation of the intervention, intervention counsellors will fill in a patient for each counselling session conducted. It includes sections on client's AUDIT score, stage of change, action and intervention plan, handing of action plan to client and comments. Intervention counsellors will be compensated with R20 (US$2.6) for each counselling session conducted and monitoring form completed. In addition, study fieldworkers will be able to report to their coordinators regarding any problems they may be having in implementing the brief interventions. Regular meetings between the researchers and the project managers will allow for any problems to be resolved timeously.

#### Outcome Measures

##### Patient measures

###### Baseline

All patients (i.e. those in the treatment and control arms) will complete baseline measures which will include a Demographic Questionnaire, medical file information (HIV status, body weight, TB treatment outcome), a health status measure, namely, the SF-12, the AUDIT to measure alcohol use/misuse, anti-tuberculosis medication adherence and two questions which ask about tobacco use.

###### Follow-up

The same measures as detailed above will be administered (at follow-up) to the intervention and control groups of patients at 3 months and again at 6 months after the baseline measures are obtained.

Broadly speaking then, the primary outcome measure of the study is: (1) change in the mean score on the AUDIT in the last 3 months and mean number of heavy drinking days in the last month of the study period compared with baseline, as measured by the AUDIT.

The secondary outcome measures of the study are: (1) anti-tuberculolisis treatment adherence and (2) successful TB response, classified by WHO as cured or treatment completed (versus treatment failure, defaulted, died or transferred out to another health facility [34].

### Sample size calculation

A total of 2600 new TB patients will be eligible and consent to be screened for alcohol misuse over a period of six months. Assuming that 20% screen positive for problem drinking, 520 new TB patients will enter the study. A minimum of 13 patients will be recruited from each of the 42 primary care clinics. It is expected that 10% of participants may be lost prior to completing the 3-months and 6-months follow-up assessments so that the final sample would be 468.

Based on the current AUDIT score of 12 among TB patients, it is assumed that the intervention will reduce the current AUDIT score by 12% to 10.6 [32,33]. Based on this assumption and taking account of the design effect of 2 to account for intraclass correlations expected from participants from the same area and followed up over time the estimated sample size of 452 will allow us with 80% power (5% level of significance) to detect the difference of 12% between the two groups.

### Data analyses

Cluster-specific methods of data analysis will be used because we will randomize clinics rather than patients.

#### Intention to treat

The principle of the statistical analysis will be intention to treat. It will be applied to the two hierarchical levels of the trial: the clinics randomized at the cluster level and the patients recruited within each of these units.

##### Clinic level

All units randomized will be included in the analysis. Units will be analyzed according to the intervention group allocated at randomization. The stratification used for the randomization of clinics (TB caseload and type of facility) will be taken into account in the analysis.

##### Patient level

All patients recruited to the study will be used in the analysis of the primary and secondary outcomes. Patients will be analyzed as a member of the clinic in which he/she was recruited.

#### Analysis approach

The primary outcome will be measured at three time points: baseline, three and at six months. If a patient drops out, is not present on the day of the interview or refuses to answer questions the primary outcome at the end point of the trial will be missing. Therefore, except for the baseline measurement, no post-randomization information will be available for these participants. The extent of the missing component was estimated to be 10% at six months. The single follow-up measurement and the extent of the expected dropout has to be considered for the statistical method that can be used to provide an unbiased estimate of the intervention effect under the principle of intention to treat. The method that can be used to take account of the stratified cluster trial design, the repeated binary nature of the primary and secondary outcome (Risky drinking, TB treatment adherence, TB treatment outcome) and the missing data at follow-up is a weighted generalized estimation equations (WGEE) approach [35]. The method utilizes inverse probability weighting to correct for the bias associated with missing data that is not missing completely at random (MCAR) and participants with complete data are weighted with the dropout probability of participants with the same characteristics at baseline. To determine the weights a logistic regression model will be constructed for the dropout indicator utilizing measurements on the primary outcome (at baseline) and other variables taken at baseline or post-randomization. For comparison and checking an unweighted analysis will also be done.

#### Reporting

Estimated treatment effects (odds ratio and incidence rate ratio) will be reported with 95% confidence intervals.

Descriptive statistics will be calculated for baseline and follow-up. No significance tests will be performed at baseline.

#### Ethical and Research Governance Approval

We have received ethical approval from the Human Sciences Research Council Research Ethics Committee (Protocol REC No.1/16/02/11). The Department of Health in South Africa has also provided approval for this study.

#### Project Timescales

The study will run for a period of 15 months beginning in March/April 2011 to June/July 2012.

## Discussion

The results of this cluster randomized trial will assist the Department of Health in South Africa decide on a health service policy that is underpinned by comprehensive approach to reduce alcohol consumption among patients with active TB. Ultimately, if this intervention model for alcohol reduction is successful, the cure rates for TB will be closer to the Strategic Plan to contain TB as a communicable disease which is highly contagious, and which at this point is still considered a "high burden disease", and with HIV co-infection HIV/TB are considered to have "double disease burden".

## List of abbreviations used

AUDIT: Alcohol Use Disorders Identification Test; CAGE: Cut Down, Annoyed, Guilty and Eye Opener (alcohol use disorders screening test); IMB: Information-Motivation-Behavioural Skills Model; NIAAA: National Institute on Alcohol Abuse and Alcoholism; SBI: Screening and Brief Intervention; TB: Tuberculosis; WHO: World Health Organisation

## Competing interests

The authors declare that they have no competing interests.

## Authors' contributions

KP and PN were the main contributors to the conceptualization of the study. KP and PN also contributed significantly to the first draft of the paper and all authors contributed to subsequent drafts and finalization. All authors read and approved the final manuscript.

## Pre-publication history

The pre-publication history for this paper can be accessed here:

http://www.biomedcentral.com/1471-2458/11/394/prepub
